# The 2011 Famine in Somalia: lessons learnt from a failed response?

**DOI:** 10.1186/1752-1505-7-22

**Published:** 2013-10-30

**Authors:** Andrew Seal, Rob Bailey

**Affiliations:** 1 UCL Institute for Global Health, Institute of Child Health, University College London, 30 Guilford Street, London WC1N 1EH, UK; 2 The Royal Institute of International Affairs, Chatham House, 10 St James’s Square, London SW1Y 4LE, UK

**Keywords:** Somalia, Famine, Early warning systems, Humanitarian action, Political context, Cluster approach, War on terror

## Abstract

**Background:**

Famine early warning systems clearly identified the risk of famine in South Central Somalia in 2010–2011 but timely action to prevent the onset of famine was not taken. The result was large scale mortality, morbidity, and population displacement.

**Discussion:**

The main factor that turned a drought-related food crisis into a famine was the war that afflicted southern Somalia and the tactics adopted by the various belligerents. These included non-state actors, regional, and international governments. In disasters and complex emergencies, such as this, we posit that five conditions need to be in place to enable humanitarian agencies to provide a timely response to early warnings of famine. These are: presence; access; adequate funding; operational capacity; and legal protection for humanitarian action. In the run up to the Somalia famine each of these presented severe challenges to humanitarian action. The design of the current coordination and funding system contributed to the problems of achieving a neutral, independent, and effective humanitarian response.

**Summary:**

The 2011 famine in Somalia was predicted and could have been mitigated or prevented if the humanitarian response had been timely and more effective. To improve responsiveness to early warnings, action is required to better insulate the humanitarian system from political agendas. While overcoming constraints, such as lack of access, may sometimes be beyond the scope of humanitarian actors, more could be done to enhance the perceived neutrality of parts of the humanitarian system. This should include a reappraisal of the cluster coordination system and reforms to donor funding mechanisms.

## Background

On 20 July 2011, in the wake of 11 months of escalating warnings, the UN declared famine in two regions of South Central Somalia [1]. Further declarations followed in four more areas over the course of the next two months [2]. The famine is thought to have cost the lives of 258,000 people, while hundreds of thousands more fled across the border into Kenya and Ethiopia [3]. Although the media focussed on drought as the main cause, the 2011 Somalia famine was caused by multiple factors that included conflict, the use of anti-terrorism legislation by the US government to prevent aid reaching Southern Somalia, an increase in global food prices, and other long-standing, structural factors [4] Additional file 1.

Early warnings of the impending health catastrophe were sufficient, timely, and robust [5-7]. What was lacking was *timely action* and an effective response from national authorities and the international humanitarian system. Various reasons for this inappropriate use of early warning information by donors and decision makers have been discussed, including their problems in dealing with the uncertainty inherent in probabilistic analysis and the absence of definitive statements about future mortality. Other factors included a lack of advocacy activity to highlight the impending crisis and the complex political environment surrounding the conflict [6].

Had there been a more effective response to early warning, then preventive interventions could have been undertaken to minimise excess mortality and morbidity. But no scale-up occurred until famine was declared, when it was, by definition, too late.

More than a year since the end of the famine was declared, food security in Somalia remains precarious and conflict continues in the south despite the transition from the previous Transitional Federal Government (TFG) to an elected parliament. But, there is now a window for reflection, which has been used by other authors to consider various aspects of the failed response and its context [8]. Here, we focus on why early warnings were ignored and what reforms to the humanitarian system are required to help prevent a recurrence of famine in Somalia or elsewhere?

## Discussion

### Analysis of the response

We posit that five conditions need to be in place for a timely response to early warnings by humanitarian agencies. These are: *presence; access; adequate funding; operational capacity;* and *legal protection* for humanitarian action. Below, we consider each in the run up to the 2011 Somalia famine.

#### Presence - the physical presence of a humanitarian agency, or its partners, in an area where humanitarian need exists or is expected to arise

Without presence, timely action in response to early warning will be difficult to achieve. In the case of Somalia key humanitarian actors were missing. The World Food Programme (WFP) is the UN agency primarily responsible for the provision of food assistance and the co-leader of the ‘Food Security Cluster’ in Somalia. As such, it is the UN ‘provider of last resort’ with an obligation to do everything it can to ‘ensure an adequate and appropriate response’ [9]. However, WFP had withdrawn from South Central Somalia in January 2010 [10]. Already suffering repeated attacks on its staff, WFP’s presence became untenable when a 2009 UN monitoring exercise released preliminary information about significant food aid diversions, including to al Shabaab and other armed opposition groups. These findings attracted a good deal of attention from US officials and the media [11]. The implementation of US legislation (discussed below) and associated pressures were other important factors that may have contributed to the decision by WFP to withdraw.

#### Access - the ability to access areas and populations in need, usually gained via the acceptance of agency activities by all major belligerents on the ground

In Somalia, humanitarian access has been challenged for decades. Humanitarian aid has formed a critical part of the economy and political power has been built upon it and used to control access to it [12]. In South Central Somalia access was denied to a number of key agencies by al Shabaab. Shortly after WFP had suspended operations in the region in 2010, it was accused of political motives and banned, making it impossible for it to return as famine approached. A further 16 UN agencies and international NGOs were later banned for “illicit activities and misconduct” during November 2011, while the famine was on-going [13]. Nonetheless, operational agencies such as Médecins San Frontières, Somalia Red Crescent Society, Islamic charities, and the International Committee of the Red Cross continued to enjoy access permissions in al-Shabaab administered areas, albeit with certain restrictions, before and during the famine. Importantly, these agencies operated largely outside of the UN-led cluster system [5,7] Additional file 2.

#### Operational capacity - the ability of a humanitarian agency to provide the level of services necessary to meet humanitarian need within its geographical area of operation and its sector of activities

Without doubt, Somalia presents a very challenging operating environment and most agencies struggle to maintain adequate human resource and material capabilities to meet the high level of need. During 2011, it was particularly difficult to maintain or build adequate operational capacity in South Central Somalia or to control or monitor the quality of relief programmes. Due to insecurity, even agencies that had access to field sites in Somalia had to usually rely on managing projects remotely from offices in Nairobi.

#### Adequate funding - the ability of a humanitarian agency to access sufficient funds in good time, so as to meet the assessed needs within its geographical area of operation and its sector of activities

Humanitarian funding in Somalia has varied greatly in recent decades, in response to both changes in need and the political priorities of donor states [12]. Funding for South Central Somalia declined by half between 2008 and 2011 as the USA withdrew support and imposed highly stringent reporting restrictions as part of efforts to prevent the use of aid by al Shabaab [14]. The EU also scaled back funding to South Central Somalia, to the extent that some member states were accused of ‘wilful neglect’ as famine struck [15].

Arguably due to the slow response of western donors, several new stakeholders did enter the donor pool for Somalia during 2011. These included Saudi Arabia, Brazil, Turkey, the Organisation for Islamic Cooperation, and China [12]. But their contributions, although substantial, were not adequate to address the shortfall.

#### Legal protection - the extent to which an agency is free to operate in a manner consistent with internationally accepted humanitarian principles without the perceived or real threat of legal action against it or its staff

Fear of litigation by governments reduced the speed and extent of responses that could have prevented the development of famine. In particular, sanctions imposed by the US Office of Foreign Assets Control and extensions to the criminal code made under the PATRIOT Act, introduced wide spread concern within the humanitarian community that organisations or individuals could be prosecuted under US law for undertaking humanitarian work in areas administered by entities labelled by the US government as ‘Foreign Terrorist Organizations’, such as al Shabaab [14,16].

### The political context

The humanitarian system failed to prevent famine in South Central Somalia in 2011, not because of a failure in early warning, but rather because the five requirements outlined above were not met. The reasons why are numerous and complex, however we argue that politics was a key factor.

The strategy of western donors in Somalia was primarily shaped by the ‘global war on terror’; the priority being to undermine al Shabaab, the *de facto* administration in the worst-affected areas. Inadequate funding was a direct and inevitable consequence of donor anti-terror legislation. So was the failure to provide an enabling legal environment for humanitarian agencies to operate without the threat of prosecution. This strategy also had serious consequences for the presence, operational capacity and access of agencies on the ground. Donor concerns about the diversion of food aid to al Shabaab almost certainly contributed to WFP’s decision to withdraw following the critical UN monitoring report. Association with western donors made it dangerous for agencies to maintain operational capacity in al Shabaab controlled areas and made al Shabaab’s decision to ban WFP and 16 other UN agencies and international NGOs more likely.

Neither was the prevention of famine of primary concern to al Shabaab. In addition to its decision to limit humanitarian access as part of its propaganda campaign against the West, reports indicate that al Shabaab also placed restrictions on the movement of people attempting to flee affected areas, and extracted agricultural taxes likely to have exacerbated food insecurity. The military campaign against the TFG and its regional and western allies was the overriding priority.

The objectives of regional powers within Somalia are complex and not purely humanitarian. For example, there are advantages to Kenya and Ethiopia in being allies of the West in the global war on terror, most obviously in terms of aid receipts. In addition, military operations in Somalia may help achieve wider economic objectives associated with the development of the Lamu corridor (also known as LAPSSET), an ambitious project which includes running an oil pipeline from Lamu on the coast of Kenya through to the oilfields of Southern Sudan [17]. Associated infrastructure and tourism development will also benefit Ethiopia. In addition, there are significant gas reserves in the Lamu basin, which lies just south of the Kenyan/Somali border.

All of these offer substantial opportunities for developing the economies of East Africa. However, the infrastructure development also requires security and a pacified Somalia, as land based incursions into the North Eastern Province of Kenya would threaten the development of the pipeline whilst marine raids could jeopardise both the development of the gas concessions and the later export of oil and gas from Lamu, only 60 miles from the border.

From this standpoint, the military operations of regional powers within Somalia, particularly those of Kenya, can be more easily understood. International attempts to blockade al Shabaab held territories were a strategy to weaken al Shabaab, but made famine in these areas more likely. The primary objective of an incursion by Kenyan troops during the famine was not to respond to the kidnapping of western tourists as originally claimed, but probably to annex the land west of the Juba river in order to create an effective buffer territory (Jubaland) [18,19].

And what of the humanitarian agencies operating in this highly complex and politicised environment? Once famine was declared they responded rapidly. However until then, agencies had collectively failed to raise the alarm or increase their consolidated appeal, on the basis that it was politically unrealistic to do so given donor policies towards Somalia [20]. Agencies also failed to collectively adapt to WFP’s absence from South Central Somalia: contingency plans were not developed despite the collapse in presence and operational capacity that this represented [5], and WFP remained the provider of last resort despite its questionable ability to perform this role whilst operationally absent from the areas most at risk.

Whilst al Shabaab’s claim that the agencies it banned were pursuing ‘illicit activities’ was probably nonsense, its underlying concern that they were somehow linked to hostile governments was not. Agencies working through the cluster system could never hope to be perceived as neutral. The cluster system was led by the UN, which in Somalia had a dual humanitarian and *political* mandate. It was heavily dependent upon western donors for its funding and had links both to the UN-mandated AMISOM force fighting al Shabaab, and the TFG. It is no surprise that those agencies operating largely outside of the cluster system maintained the greatest access.

### Conclusions and recommendations

Macrae and Zwi distinguish between acts of omission and acts of commission when evaluating how the actions of warring parties may lead to hunger or famine [21]. Whilst the facts that fully explain who did what and why may never come to light, *prima facie* it appears that the 2011 Somalia famine followed from multiple acts of politically motivated omission and commission. Al Shabaab expelled humanitarian agencies. Donor governments withheld funding despite increasingly urgent warnings of impending famine. Regional powers undertook military operations that made famine more, not less likely. Fundamentally, the various political agendas of donor governments, regional powers, and the warring authorities within Somalia were incompatible with the prevention of famine and hindered the ability of the UN-led cluster system to operate independently and effectively.

## Summary

What can be done to better insulate the humanitarian system from political influences and prevent these failures from being repeated? The interface between agencies and donor governments must be better delineated and scrutinised. In particular, arrangements that can help isolate funding decisions from geopolitical agendas must be explored. This might include a greater use of pooled funds into which donors pay regular upfront contributions, devolving allocation decisions to agencies to be based on need. Innovative financing mechanisms which release funds according to pre-agreed early warning triggers, allowing agencies to access early funding without recourse to political decision-making, may also offer some potential. At a minimum, donor governments should develop clear and transparent guidelines that specify when particular humanitarian interventions are warranted and on what basis they will fund them, so that they can be held accountable to these Additional file 3.

The cluster system, embedded within the UN and tied to national governments, offers important advantages in situations where the political agendas of donors and national authorities are aligned. However in complex emergencies such as Somalia, characterised by conflict and multiple opposing political agendas, there is a fundamental mismatch between the design of the cluster system and the need to achieve both actual and perceived humanitarian neutrality. A rethink is needed.

The reforms outlined above are ambitious, if not aspirational. They would certainly be resisted by donor governments standing to lose power in a depoliticised system. Nonetheless, they are needed to reduce the risk of the 2011 famine being repeated, in Somalia or in a complex emergency elsewhere.

## Abbreviations

AMISOM: African Union Mission in Somalia; FAO: Food and Agriculture Organization; IASC: Inter-Agency Standing Committee; ICU: Islamic Courts Union; TFG: Transitional Federal Government; UN: United Nations; US: United States; WFP: World Food Programme.

## Competing interests

Both authors declare that they have no competing interests.

## Authors’ contributions

AS conceived and wrote the initial draft. RB researched and revised it, making substantial changes. Both authors read and approved the final manuscript.

## Authors’ information

AS has worked in international nutrition for the last 15 years; conducting research in emergency and protracted refugee situations. He was the lead technical advisor on the national micronutrient survey in Somalia in 2009 and the UNHCR survey of famine-affected Somali refugees in the Dadaab camps in 2011.

RB is a Senior Research Fellow at Chatham House leading on food security. Prior to this he worked for Oxfam in a variety of roles. He is currently leading a major research project on the barriers to translating early warning into early action for slow onset food crises, funded by the Rockefeller Foundation and the Disasters Emergency Committee.

## Supplementary Material

Additional file 1 **Background to Somalia **[22,28].
Click here for file

Additional file 2 **The Cluster Approach for Coordinating Humanitarian Response to Disasters and Complex Emergencies **[29].
Click here for file

Additional file 3 **Humanitarian Principles and Humanitarian Action **[30,31].
Click here for file
