# Supplementary material for: The 2011 Famine in Somalia: lessons learnt from a failed response?
Source: Confl Health. 2013 Oct 30;7:22. doi: 10.1186/1752-1505-7-22 (PMC3829375; doi:10.1186/1752-1505-7-22)
Supplement: Additional file 1 — Background to Somalia [22,28]. [file 1752-1505-7-22-S1.doc]

**Additional file 1: Background to Somalia.**

| Formed in 1960, Somalia is a relatively young state, and has been without a strong central government since the fall of the Siad Barre administration in 1991.  There are three autonomous or semi-autonomous regions within Somalia: Somaliland, Puntland, and South Central Somalia. The Republic of Somaliland declared itself independent in 1991, but this declaration has not been recognised by other states or the current central government in Mogadishu [22,23]. In addition to regional political dynamics, clan affiliations play a vital role in the social and political life of Somalia.  Somalia has been conflict-affected for much of its history. A war for possession of the Ogaden region took place between Somalia and Ethiopia between 1977 and 1978. Following the end of hostilities with Ethiopia a civil war badly affected the country and led to the famine of 1991–1992.  There has been a long history of intervention from foreign states, with, for example, both the USSR and the US supporting the government in Mogadishu at different times. The US military intervention in 1992 and its targeting of political leader Mohamed Farah Aideed led to the infamous ‘black hawk down’ incident [24].  Within this context, humanitarian aid has been used as an important economic and political tool for decades. As a result, there has been a mixed adherence to the principles of humanitarian action and an erosion of trust and humanitarian space [12].  Following 9/11, the US ‘global war on terror’ has shaped strategies towards Somalia, in particular focussing efforts on the establishment of a western friendly central government. Formed in 2004, the Transitional Federal Government (TFG) governed impotently from neighbouring Kenya, whilst Somalia came to be governed by a group of Sharia courts known as the Islamic Courts Union (ICU). In 2006, US-backed Ethiopian military action to remove the ICU allowed the TFG to relocate to Somalia. However, the TFG remains weak and relies on support from both western and regional powers.  With the defeat of the UIC, the offshoot ‘Islamist’ group, al Shabaab, rose to the fore in South Central Somalia and became the effective administration in most areas by 2008. It controlled parts of Mogadishu until August 2011, when it was forced to withdraw due to military pressure from forces deployed by the UN-mandated African Union Mission in Somalia (AMISOM).  Even after withdrawing from Mogadishu, al Shabaab continued to govern the majority of Southern Somalia, both before and during the famine of 2011–12. However, when Kenyan troops invaded Southern Somalia during the peak of the famine in October 2011, al Shabaab started to lose ground, eventually being driven out of the key regional port city of Kismayo in October, 2012.  Ugandan, Djiboutian, and Kenyan troops are all involved in overt military action inside Somalia under the banner of AMISOM, while Ethiopian troops operate outside of the UN mission. In addition, the USA and other states have been engaged in covert military action to assist the former TFG and its allies [25].  The TFG’s mandate expired on 20 August 2012, and while a new government has formed in Mogadishu, discussions continue over the contents and meaning of the draft national constitution [26].  Conflict continues over control of the ‘Jubaland’ region of South Central Somalia and the key port of Kismayo. As of June 2013, fighting is on-going between the Kenyan-backed Ras Kamboni Brigade and other armed groups [27]. Large parts of rural Southern Somalia remain under control of al Shabaab, and al Shabaab attacks on high profile political targets in Mogadishu continue to occur. Despite seeing some grounds for optimism, the prospects for a unified and peaceful Somalia currently appear remote [28]. |
| --- |
