# Supplementary material for: The 2011 Famine in Somalia: lessons learnt from a failed response?
Source: Confl Health. 2013 Oct 30;7:22. doi: 10.1186/1752-1505-7-22 (PMC3829375; doi:10.1186/1752-1505-7-22)
Supplement: Additional file 2 — The Cluster Approach for Coordinating Humanitarian Response to Disasters and Complex Emergencies [29]. [file 1752-1505-7-22-S2.doc]

**Additional file 2: The Cluster Approach for Coordinating Humanitarian Response to Disasters and Complex Emergencies.**

| The Humanitarian Reform process was initiated in 2005 by Jan Egeland, the then UN Emergency Relief Coordinator, together with the Inter-Agency Standing Committee (IASC), the coordination forum for major international UN and non-UN humanitarian agencies. The aim of the process was to “*improve the effectiveness of humanitarian response through greater predictability, accountability, responsibility and partnership*”.  Central to this reform was the cluster approach, based upon the organisation of humanitarian agencies into 11 distinct technical ‘clusters’ at the global level. Each has a designated lead agency (almost always from the UN) responsible for standards setting, building response capacity and providing operational support. For example, the Global Nutrition Cluster is led by UNICEF whilst the Global Health cluster is led by WHO.  The cluster approach is replicated within countries experiencing humanitarian emergencies. Agencies are organised into technical clusters, with lead agencies that mirror the global leads. However, flexibility is allowed. For example, where the capacity of a global lead agency is limited at the national level, then a different lead agency can be selected. A key responsibility of a lead agency at the national level is to act as the ‘provider of last resort’. As such the agency has certain responsibilities: “*Where necessary, and depending on access, security and availability of funding, the cluster lead, as provider of last resort, must be ready to ensure the provision of services required to fill critical gaps identified by the cluster.”*  In Somalia, the Food Assistance Cluster (led by WFP) and the Agriculture and Livelihoods Cluster (led by the UN Food and Agriculture Organization (FAO)) were merged to form the Food Security Cluster, which was active in Somalia during the 2011 famine, and was co-led by both [9,29]. |
| --- |
