# Supplementary material for: The 2011 Famine in Somalia: lessons learnt from a failed response?
Source: Confl Health. 2013 Oct 30;7:22. doi: 10.1186/1752-1505-7-22 (PMC3829375; doi:10.1186/1752-1505-7-22)
Supplement: Additional file 3 — Humanitarian Principles and Humanitarian Action [30,31]. [file 1752-1505-7-22-S3.doc]

**Additional file 3: Humanitarian Principles and Humanitarian Action.**

| Humanitarian principles are based on the foundation that people affected by disasters have a right to receive assistance. Such assistance should be delivered such that it is *neutral* (does not favour any side in a conflict), *impartial* (based solely on humanitarian need, without discrimination between or within affected populations) and *independent* (autonomous from political, economic or other objectives). Adherence to these principles is viewed as a pragmatic, operational approach aimed at allowing organisations and individuals involved in delivering aid to remain outside of on-going conflict, obtain and retain the trust of parties to the conflict, and thereby gain access to vulnerable civilian populations.  The legal basis of humanitarian action is enshrined in various branches of international law, and in conflict situations is underpinned by the Geneva Conventions of 1949 and Additional Protocols of 1977. At the operational level, the Sphere Project, a voluntary initiative among humanitarian agencies, sets out a rights-based approach to humanitarian assistance, detailing what disaster affected people can expect to receive from those providing assistance. This approach is itself founded on the Sphere Humanitarian Charter and the Code of Conduct for the International Red Cross and Red Crescent Movement and Non-Governmental Organisations in Disaster Relief [30]. Finally, the guiding principles of neutrality, impartiality and independence are also enshrined in the Principles of Good Humanitarian Donorship, to which 37 major donor governments are committed [31]. |
| --- |
